# Care of the Terminally Ill Patient in India: Comments on the Proposed Legislation

**DOI:** 10.1200/JGO.2016.006858

**Published:** 2016-10-05

**Authors:** Aju Mathew, M. R. Rajagopal

**Affiliations:** **Aju Mathew**, University of Kentucky, Lexington, KY; **M. R. Rajagopal**, Pallium India, Arumana Hospital, Thiruvananthapuram, India

## TO THE EDITOR:

The Government of India recently published a draft version of the Medical Treatment of Terminally-Ill Patients Bill for comments and feedback from the public.^1^ The bill, when enacted, will provide some clarity to end-of-life care in terminally ill patients in India and is intended to protect both physicians and patients from potential liability. Such a law is relevant for the global oncology community because few low- or middle-income countries have enacted similar legislation.^2^ It may pave the way for enactment of similar legal directives in other developing countries, thereby resulting in a greater impact around the globe.

The legislation provides clarity to several facets of end-of-life care. Competent patients can decline introduction of life-sustaining medical treatment, a decision with which their physicians will be bound to comply. The legislation seeks to protect both the patient and the physician from legal complications that may arise from such a decision. It also emphasizes the importance of appropriate documentation and record keeping.

Although the Government of India must be applauded for moving ahead with this legislation, we believe that the current version may be a misstep for two reasons. First, the proposed bill prohibits the use of advance medical directives—living wills or medical powers of attorney—in India. Therefore, any advance medical directive prepared by a patient with a terminal illness for his or her health care providers and family in the event he or she becomes unable to take a decision about life-sustaining treatment will not be legally binding. This will result in increased use of life-sustaining treatment and intensive care unit admissions among terminally ill patients for whom death is an inevitable event. We fear this violates the autonomy of the patient. End-of-life discussions can reduce the rates of intensive care unit admissions and life-sustaining treatment use, such as the use of a ventilator, at the end of life.^3^ Families of terminally ill patients have also reported less stress and greater satisfaction with care after such discussions.^4^ If the principle of patient autonomy is to be respected, the proposed bill should consider upholding advance directives as an integral part of end-of-life care of terminally ill patients.

Second, it is unreasonable to expect the court to take decisions on withholding or withdrawing life-sustaining care from terminally ill patients who are unable to make informed decisions. The Indian legal system is already burdened by backlogged cases, and such an expectation could potentially harm the care of terminally ill patients by causing unnecessary delays. Instead, the bill should consider providing guidance to the formation of hospital-based ethics committees comprising physicians from various specialties, including palliative care, nurses, and representatives from the community, who could decide on the ethical aspects of withholding or withdrawing life-sustaining medical care.

In summary, we congratulate the Government of India on its noble intention of creating legislation defining appropriate end-of-life care. We hope to bring attention to the potential negative impact of the proposed bill on patient autonomy with regard to advance medical directives.

## References

[1] Republic of India http://www.prsindia.org/uploads/media//draft/Draft%20Passive%20Euthanansia%20Bill.pdf.

[2] Blank RH (2011). End-of-life decision making across cultures. J Law Med Ethics.

[3] Wright AA, Zhang B, Ray A (2008). Associations between end-of-life discussions, patient mental health, medical care near death, and caregiver bereavement adjustment. JAMA.

[4] Detering KM, Hancock AD, Reade MC (2010). The impact of advance care planning on end of life care in elderly patients: Randomised controlled trial. BMJ.

